# HDDM: Hierarchical Bayesian estimation of the Drift-Diffusion Model in Python

**DOI:** 10.3389/fninf.2013.00014

**Published:** 2013-08-02

**Authors:** Thomas V. Wiecki, Imri Sofer, Michael J. Frank

**Affiliations:** Department of Cognitive, Linguistic and Psychological Sciences, Brown UniversityProvidence, RI, USA

**Keywords:** Bayesian modeling, drift diffusion model, Python, decision-making, software

## Abstract

The diffusion model is a commonly used tool to infer latent psychological processes underlying decision-making, and to link them to neural mechanisms based on response times. Although efficient open source software has been made available to quantitatively fit the model to data, current estimation methods require an abundance of response time measurements to recover meaningful parameters, and only provide point estimates of each parameter. In contrast, hierarchical Bayesian parameter estimation methods are useful for enhancing statistical power, allowing for simultaneous estimation of individual subject parameters and the group distribution that they are drawn from, while also providing measures of uncertainty in these parameters in the posterior distribution. Here, we present a novel Python-based toolbox called HDDM (hierarchical drift diffusion model), which allows fast and flexible estimation of the the drift-diffusion model and the related linear ballistic accumulator model. HDDM requires fewer data per subject/condition than non-hierarchical methods, allows for full Bayesian data analysis, and can handle outliers in the data. Finally, HDDM supports the estimation of how trial-by-trial measurements (e.g., fMRI) influence decision-making parameters. This paper will first describe the theoretical background of the drift diffusion model and Bayesian inference. We then illustrate usage of the toolbox on a real-world data set from our lab. Finally, parameter recovery studies show that HDDM beats alternative fitting methods like the χ^2^-quantile method as well as maximum likelihood estimation. The software and documentation can be downloaded at: http://ski.clps.brown.edu/hddm_docs/

## Introduction

Sequential sampling models (SSMs) (Townsend and Ashby, 1983) have established themselves as the de-facto standard for modeling response-time data from simple two-alternative forced choice decision making tasks (Smith and Ratcliff, 2004). Each decision is modeled as an accumulation of noisy information indicative of one choice or the other, with sequential evaluation of the accumulated evidence at each time step. Once this evidence crosses a threshold, the corresponding response is executed. This simple assumption about the underlying psychological process has the appealing property of reproducing not only choice probabilities, but the full distribution of response times for each of the two choices. Models of this class have been used successfully in mathematical psychology since the 60's and more recently adopted in cognitive neuroscience investigations. These studies are typically interested in neural mechanisms associated with the accumulation process or for regulating the decision threshold (e.g., Forstmann et al., 2008; Ratcliff et al., 2009; Cavanagh et al., 2011). One issue in such model-based cognitive neuroscience approaches is that the trial numbers in each condition are often low, making it difficult to estimate model parameters. For example, studies with patient populations, especially if combined with intra-operative recordings, typically have substantial constraints on the duration of the task. Similarly, model-based fMRI or EEG studies are often interested not in static model parameters, but how these dynamically vary with trial-by-trial variations in recorded brain activity. Efficient and reliable estimation methods that take advantage of the full statistical structure available in the data across subjects and conditions are critical to the success of these endeavors.

Bayesian data analytic methods are quickly gaining popularity in the cognitive sciences because of their many desirable properties (Kruschke, 2010; Lee and Wagenmakers, 2013). First, Bayesian methods allow inference of the full posterior distribution of each parameter, thus quantifying uncertainty in their estimation, rather than simply provide their most likely value. Second, hierarchical modeling is naturally formulated in a Bayesian framework. Traditionally, psychological models either assume subjects are completely independent of each other, fitting models separately to each individual, or that all subjects are the same, fitting models to the group as if they are all copies of some “average subject.” Both approaches are sub-optimal in that the former fails to capitalize on statistical strength offered by the degree to which subjects are similar with respect to one or more model parameters, whereas the latter approach fails to account for the differences among subjects, and hence could lead to a situation where the estimated model cannot fit any individual subject. The same limitations apply to current DDM software packages such as DMAT (Vandekerckhove and Tuerlinckx, 2008) and fast-dm (Voss and Voss, 2007). Hierarchical Bayesian methods provide a remedy for this problem by allowing group and subject parameters to be estimated simultaneously at different hierarchical levels (Kruschke, 2010; Vandekerckhove et al., 2011; Lee and Wagenmakers, 2013). Subject parameters are assumed to be drawn from a group distribution, and to the degree that subjects are similar to each other, the variance in the group distribution will be estimated to be small, which reciprocally has a greater influence on constraining parameter estimates of any individual. Even in this scenario, the method still allows the posterior for any given individual subject to differ substantially from that of the rest of the group given sufficient data to overwhelm the group prior. Thus the method capitalizes on statistical strength shared across the individuals, and can do so to different degrees even within the same sample and model, depending on the extent to which subjects are similar to each other in one parameter vs. another. In the DDM for example, it may be the case that there is relatively little variability across subjects in the perceptual time for stimulus encoding, quantified by the “non-decision time” but more variability in their degree of response caution, quantified by the “decision threshold.” The estimation should be able to capitalize on this structure so that the non-decision time in any given subject is anchored by that of the group, potentially allowing for more efficient estimation of that subject's decision threshold. This approach may be particularly helpful when relatively few trials per condition are available for each subject, and when incorporating noisy trial-by-trial neural data into the estimation of DDM parameters.

HDDM is an open-source software package written in Python which allows (1) the flexible construction of hierarchical Bayesian drift diffusion models and (2) the estimation of its posterior parameter distributions via PyMC (Patil et al., 2010). User-defined models can be created via a simple Python script or be used interactively via, for example, the IPython interpreter shell (Péerez and Granger, 2007). All run-time critical functions are coded in Cython (Behnel et al., 2011) and compiled natively for speed which allows estimation of complex models in minutes. HDDM includes many commonly used statistics and plotting functionality generally used to assess model fit. The code is released under the permissive BSD 3-clause license, test-covered to assure correct behavior and well documented. An active mailing list exists to facilitate community interaction and help users. Finally, HDDM allows flexible estimation of trial-by-trial regressions where an external measurement (e.g., brain activity as measured by fMRI) is correlated with one or more decision-making parameters.

This report is intended to familiarize experimentalists with the usage and benefits of HDDM. The purpose of this report is thus two-fold; (1) we briefly introduce the toolbox and provide a tutorial on a real-world data set (a more comprehensive description of all the features can be found online); and (2) characterize its success in recovering model parameters by performing a parameter recovery study using simulated data to compare the hierarchical model used in HDDM to non-hierarchical or non-Bayesian methods as a function of the number of subjects and trials. We show that it outperforms these other methods and has greater power to detect dependencies of model parameters on other measures such as brain activity, when such relationships are present in the data. These simulation results can also inform experimental design by showing minimum number of trials and subjects to achieve a desired level of precision.

## Methods

### Drift diffusion model

SSMs generally fall into one of two classes: (1) diffusion models which assume that *relative* evidence is accumulated over time and (2) race models which assume independent evidence accumulation and response commitment once the first accumulator crossed a boundary (LaBerge, 1962; Vickers, 1970). Currently, HDDM includes two of the most commonly used SSMs: the drift diffusion model (DDM) (Ratcliff and Rouder, 1998; Ratcliff and McKoon, 2008) belonging to the class of diffusion models and the linear ballistic accumulator (LBA) (Brown and Heathcote, 2008) belonging to the class of race models. In the remainder of this paper we focus on the more commonly used DDM.

As input these methods require trial-by-trial RT and choice data (HDDM currently only supports binary decisions) as illustrated in the below example table:

**Table:** 

**RT**	**Response**	**Condition**	**Brain measure**
0.8	1	hard	0.01
1.2	0	easy	0.23
0.25	1	hard	−0.3

**Figure 1:**
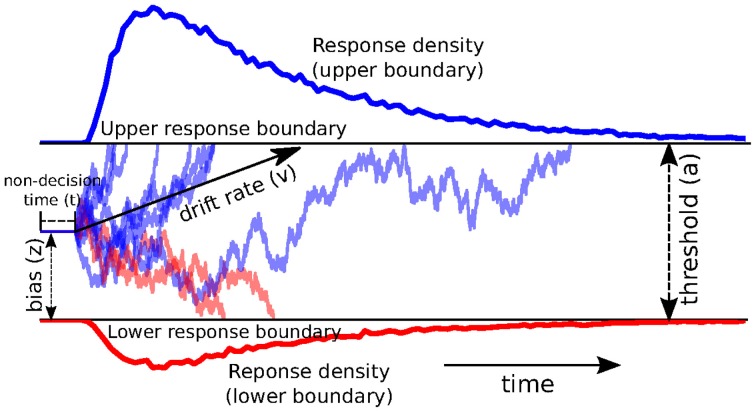
**Trajectories of multiple drift-processes (blue and red lines, middle panel)**. Evidence is noisily accumulated over time (x-axis) with average drift-rate *v* until one of two boundaries (separated by threshold *a*) is crossed and a response is initiated. Upper (blue) and lower (red) panels contain density plots over boundary-crossing-times for two possible responses. The flat line in the beginning of the drift-processes denotes the non-decision time *t* where no accumulation happens. The histogram shapes match closely to those observed in response time measurements of research participants. Note that HDDM uses a closed-form likelihood function and not actual simulation as depicted here.

The DDM models decision-making in two-choice tasks. Each choice is represented as an upper and lower boundary. A drift-process accumulates evidence over time until it crosses one of the two boundaries and initiates the corresponding response (Ratcliff and Rouder, 1998; Smith and Ratcliff, 2004) (see Figure 1 for an illustration). The speed with which the accumulation process approaches one of the two boundaries is called drift-rate *v*. Because there is noise in the drift process, the time of the boundary crossing and the selected response will vary between trials. The distance between the two boundaries (i.e., threshold *a*) influences how much evidence must be accumulated until a response is executed. A lower threshold makes responding faster in general but increases the influence of noise on decision-making and can hence lead to errors or impulsive choice, whereas a higher threshold leads to more cautious responding (slower, more skewed RT distributions, but more accurate). Response time, however, is not solely comprised of the decision-making process—perception, movement initiation and execution all take time and are lumped in the DDM by a single non-decision time parameter *t*. The model also allows for a prepotent bias *z* affecting the starting point of the drift process relative to the two boundaries. The termination times of this generative process gives rise to the response time distributions of both choices.

An analytic solution to the resulting probability distribution of the termination times was provided by Wald (1947); Feller (1968):

$$\begin{array}{l} f(x|v,a,z)=\frac{\pi }{a^{2}}\text{exp​}\left(-vaz-\frac{v^{2}x}{2}\right)\\ \;\times \sum_{k=1}^{\infty }k\text{ exp​}\left(-\frac{k^{2}\pi^{2}x}{2a^{2}}\right)\text{sin}\left(k\pi z\right) \end{array}$$

Since the formula contains an infinite sum, HDDM uses an approximation provided by Navarro and Fuss (2009).

Subsequently, the DDM was extended to include additional noise parameters capturing inter-trial variability in the drift-rate, the non-decision time and the starting point in order to account for two phenomena observed in decision-making tasks, most notably cases where errors are faster or slower than correct responses. Models that take this into account are referred to as the full DDM (Ratcliff and Rouder, 1998). HDDM uses analytic integration of the likelihood function for variability in drift-rate and numerical integration for variability in non-decision time and bias (Ratcliff and Tuerlinckx, 2002).

### Hierarchical bayesian estimation of the drift-diffusion model

Statistics and machine learning have developed efficient and versatile Bayesian methods to solve various inference problems (Poirier, 2006). More recently, they have seen wider adoption in applied fields such as genetics (Stephens and Balding, 2009) and psychology (Clemens et al., 2011). One reason for this Bayesian revolution is the ability to quantify the certainty one has in a particular estimation of a model parameter. Moreover, hierarchical Bayesian models provide an elegant solution to the problem of estimating parameters of individual subjects and groups of subjects, as outlined above. Under the assumption that participants within each group are similar to each other, but not identical, a hierarchical model can be constructed where individual parameter estimates are constrained by group-level distributions (Shiffrin et al., 2008; Nilsson et al., 2011).

**Figure 2:**
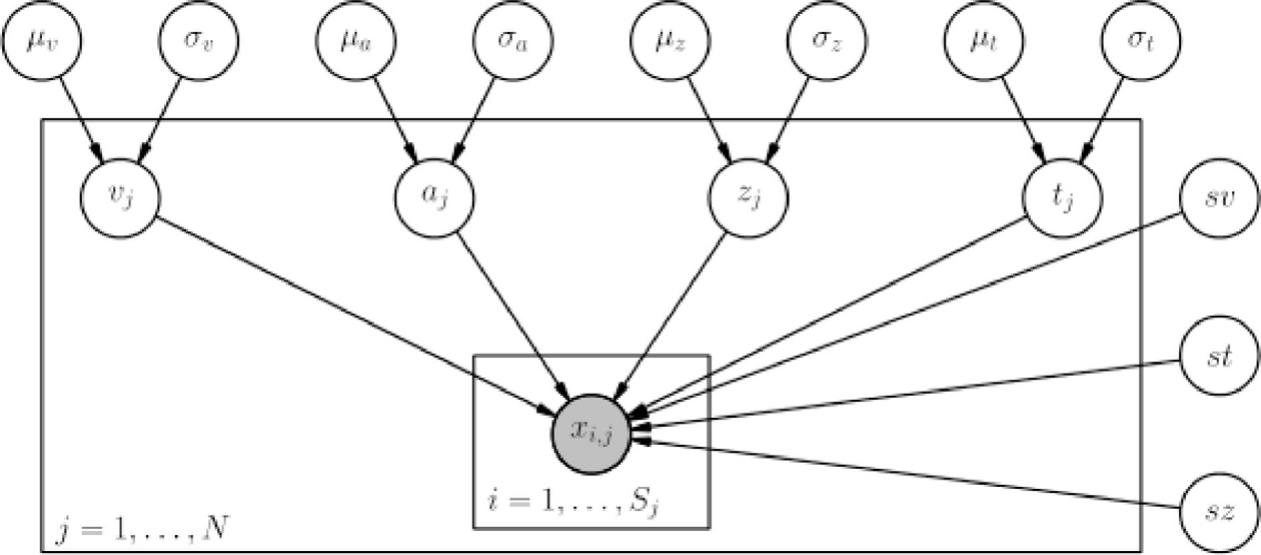
**Basic graphical hierarchical model implemented by HDDM for estimation of the drift-diffusion model**. Round nodes represent random variables. Shaded nodes represent observed data. Directed arrows from parents to children visualize that parameters of the child random variable are distributed according to its parents. Plates denote that multiple random variables with the same parents and children exist. The outer plate is over subjects while the inner plate is over trials.

HDDM includes several hierarchical Bayesian model formulations for the DDM and LBA. For illustrative purposes we present the graphical model depiction of a hierarchical DDM with informative priors and group-only inter-trial variability parameters in Figure 2. Note, however, that there is also a model with non-informative priors which the user can opt to use. Nevertheless, we recommend using informative priors as they constrain parameter estimates to be in the range of plausible values based on past literature (Matzke and Wagenmakers, 2009) (see the supplement), which can aid in reducing issues with parameter collinearity, and leads to better recovery of true parameters in simulation studies—especially with few trials as shown below.

Graphical nodes are distributed as follows: 
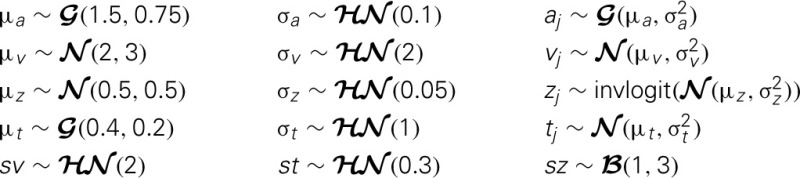


and *x*_*i, j*_ ~ *F*(*a*_*i*_, *z*_*i*_, *v*_*i*_, *t*_*i*_, *sv, st, sz*) where *x*_*i, j*_ represents the observed data consisting of response time and choice of subject *i* on trial *j* and *F* represents the DDM likelihood function as formulated by Navarro and Fuss (2009). 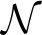 represents a normal distribution parameterized by mean and standard deviation, 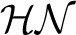 represents a positive-only, half-normal parameterized by standard-deviation, 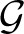 represents a Gamma distribution parameterized by mean and rate, 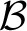 represents a Beta distribution parameterized by α and β. Note that in this model we do not attempt to estimate individual parameters for inter-trial variabilities. The reason is that the influence of these parameters onto the likelihood is often so small that very large amounts of data would be required to make meaningful inference at the individual level.

HDDM then uses Markov chain Monte Carlo (MCMC) (Gamerman and Lopes, 2006) to estimate the joint posterior distribution of all model parameters (for more information on hierarchical Bayesian estimation we refer to the supplement).

Note that the exact form of the model will be user-dependent; consider as an example a model where separate drift-rates *v* are estimated for two conditions in an experiment: easy and hard. In this case, HDDM will create a hierarchical model with group parameters μ_*v*_easy__, σ_*v*_easy__, μ_*v*_hard__, σ_*v*_hard__, and individual subject parameters *v*_*j*_easy__, and *v*_*j*_hard__.

## Results

In the following we will demonstrate how HDDM can be used to infer different components of the decision-making process in a reward-based learning task. While demonstrating core features this is by no means a complete overview of all the functionality in HDDM. For more information, an online tutorial and a reference manual see http://ski.clps.brown.edu/hddm_docs.

Python requires modules to be imported before they can be used. The following code imports the hddm module into the Python name-space:

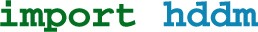



### Loading data

It is recommended to store your trial-by-trial response time and choice data in a csv (comma-separated-value, see below for exact specifications) file. In this example we will be using data collected in a reward-based decision-making experiment in our lab (Cavanagh et al., 2011). In brief, at each trial subjects choose between two symbols. The trials were divided into win-win trials (WW), in which the two symbols were associated with high winning chances; lose-lose trials (LL), in which the symbols were associated with low winning chances, and win-lose trials (WL), which are the easiest because only one symbol was associated with high winning chances. Thus WW and LL decisions together comprise high conflict (HC) trials (although there are other differences between them, we do not focus on those here), whereas WL decisions are low conflict (LC). The main hypothesis of the study was that high conflict trials induce an increase in the decision threshold, and that the mechanism for this threshold modulation depends on communication between mediofrontal cortex (which exhibits increased activity under conditions of choice uncertainty or conflict) and the subthalamic nucleus (STN) of the basal ganglia (which provides a temporary brake on response selection by increasing the decision threshold). The details of this mechanism are described in other modeling papers (e.g., Ratcliff and Frank, 2012). Cavanagh et al. (2011) tested this theory by measuring EEG activity over mid-frontal cortex, focusing on the theta band, given prior associations with conflict, and testing whether trial-to-trial variations in frontal theta were related to adjustments in decision threshold during HC trials. They tested the STN component of the theory by administering the same experiment to patients who had deep brain stimulation (DBS) of the STN, which interferes with normal processing and was tested in the on and off condition.

The first ten lines of the data file look as follows.

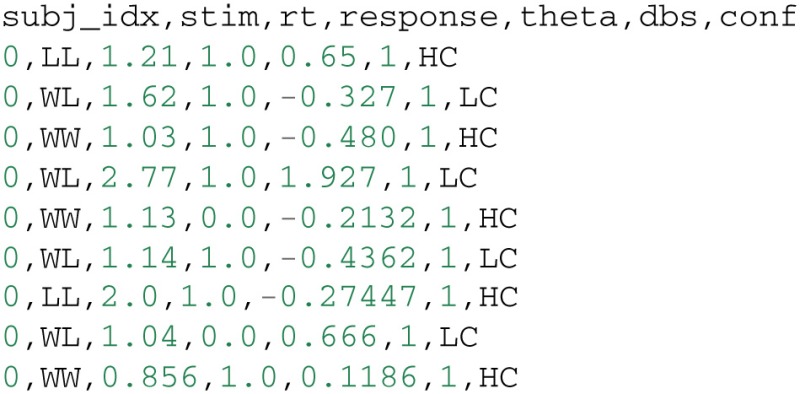



The first row represents the column names; each following row corresponds to values associated with a column on an individual trial. While subj_idx (unique subject identifier), rt (response time) and response (binary choice) are required, additional columns can represent experiment specific data. Here, theta represents theta power as measured by EEG, dbs whether DBS was turned on or off, stim which stimulus type was presented and conf the conflict level of the stimulus (see above).

The hddm.load_csv() function can then be used to load this file.

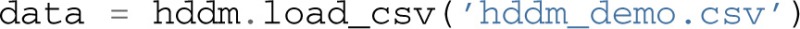



### Fitting a hierarchical model

The HDDM class constructs a hierarchical DDM that can later be fit to subjects' RT and choice data, as loaded above. By supplying no extra arguments other than data, HDDM constructs a simple model that does not take our different conditions into account. To speed up convergence, the starting point is set to the maximum a-posterior value (MAP) by calling the HDDM.find_starting_values method which uses gradient ascent optimization. The HDDM.sample() method then performs Bayesian inference by drawing posterior samples using the MCMC algorithm.

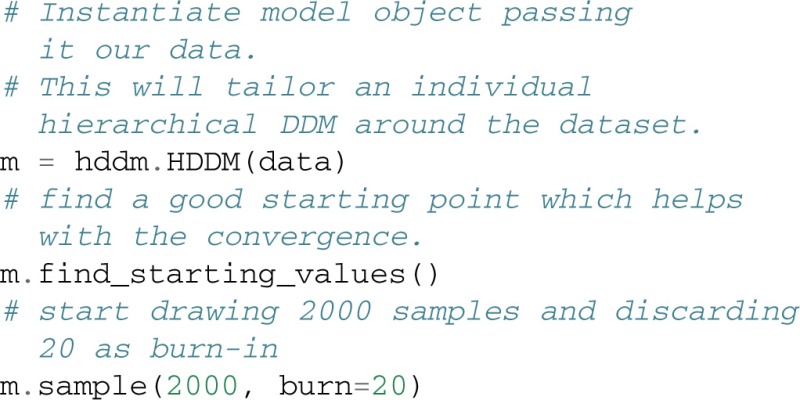



We recommend drawing between 2000 and 10,000 posterior samples, depending on the convergence. Discarding the first 20–1000 samples as burn-in is often enough in our experience. Auto-correlation of the samples can be reduced by adding the thin=n keyword to sample() which only keeps every n-th sample, but unless memory is an issue we recommend keeping all samples and instead drawing more samples if auto-correlation is high.

Note that it is also possible to fit a non-hierarchical model to an individual subject by setting is_group_model=False in the instantiation of HDDM or by passing in data which lacks a subj_idx column. In this case, HDDM will use the group-mean priors from above for the DDM parameters.

**Figure 3:**
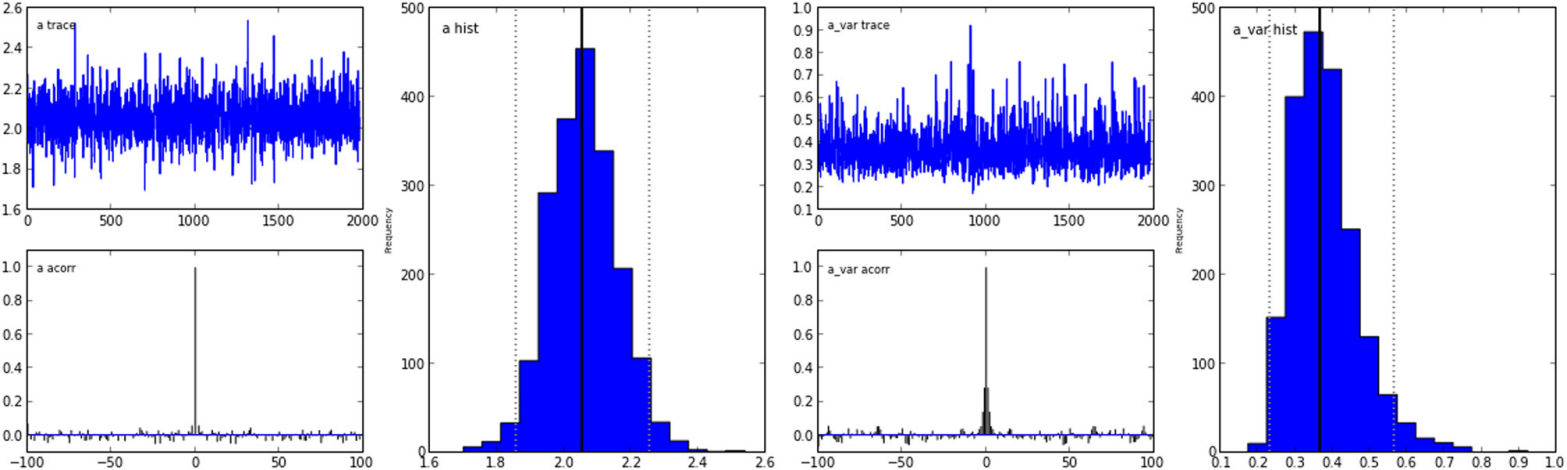
**Posterior plots for the group mean (left half) and group standard-deviation (right half) of the threshold parameter *a***. Posterior trace (upper left inlay), auto-correlation (lower left inlay), and marginal posterior histogram (right inlay; solid black line denotes posterior mean and dotted black line denotes 2.5 and 97.5% percentiles).

The inference algorithm, MCMC, requires the chains of the model to have properly converged. While there is no way to guarantee convergence for a finite set of samples in MCMC, there are many heuristics that allow identification of problems of convergence. One analysis to perform is to visually investigate the trace, the autocorrelation, and the marginal posterior. These can be plotted using the HDDM.plot_posteriors() method (see Figure 3). For the sake of brevity we only plot two here (group mean and standard deviation of threshold). In practice, however, one should examine all of them.

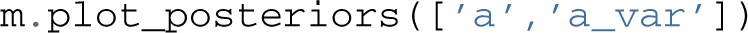



Problematic patterns in the trace would be drifts or large jumps which are absent here. The autocorrelation should also drop to zero rather quickly (i.e., well smaller than 50) when considering the influence of past samples, as is the case here.


_______________________________________
m.print_stats()
               mean       std      2.5q       25q       50q       75q  97.5q
a          2.058015  0.102570  1.862412  1.988854  2.055198  2.123046  2.261410
a_var      0.379303  0.089571  0.244837  0.316507  0.367191  0.426531  0.591643
a_subj.0   2.384066  0.059244  2.274352  2.340795  2.384700  2.423012  2.500647
_______________________________________


The Gelman-Rubin $\hat{R}$ statistic (Gelman and Rubin, 1992) provides a more formal test for convergence that compares within-chain and between-chain variance of different runs of the same model. This statistic will be close to 1 if the samples of the different chains are indistinguishable. The following code demonstrates how five models can be run in a for-loop and stored in a list (here called models).

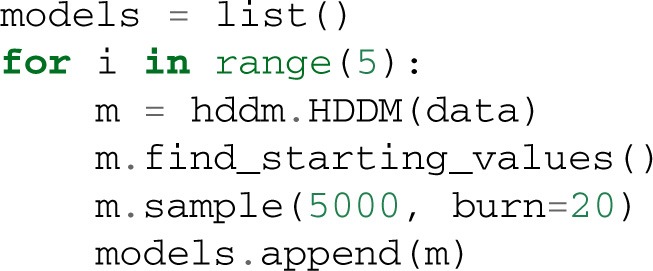




hddm.analyze.gelman_rubin(models)


Which produces the following output (abridged to preserve space): 
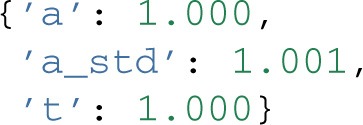


Values should be close to 1 and not larger than 1.02 which would indicate convergence problems.

Once convinced that the chains have properly converged we can analyze the posterior values. The HDDM.print_stats() method outputs a table of summary statistics for each parameters' posterior).

The output contains various summary statistics describing the posterior of each parameter: group mean parameter for threshold a, group variability a_var and individual subject parameters a_subj.0. Other parameters are not shown here for brevity but would be outputted normally.

As noted above, this model did not take the different conditions into account. To test whether the different reward conditions affect drift-rate we create a new model which estimates separate drift-rate v for the three conflict conditions. HDDM supports splitting by condition in a between-subject manner via the depends_on keyword argument supplied to the HDDM class. This argument expects a Python dict which maps the parameter to be split to the column name containing the conditions we want to split by. This way of defining parameters to be split by condition is directly inspired by the fast-dm toolbox (Voss and Voss, 2007).

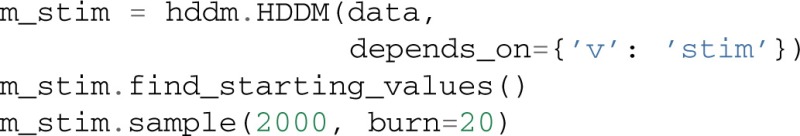



Note that while every subject was tested on each condition in this case, this is not a requirement. The depends_on keyword can also be used to test between-group differences. For example, if we collected data where one group received a drug and the other one a placebo we would include a column in the data labeled 'drug' that contained 'drug' or 'placebo' for each subject. In our model specification we could test the hypothesis that the drug affects threshold by specifying depends_on = {'a': 'drug'}. In this case HDDM would create and estimate separate group distributions for the two groups/conditions. After selecting an appropriate model (e.g., via model selection) we could compare the two group mean posteriors to test whether the drug is effective or not.

**Figure 4:**
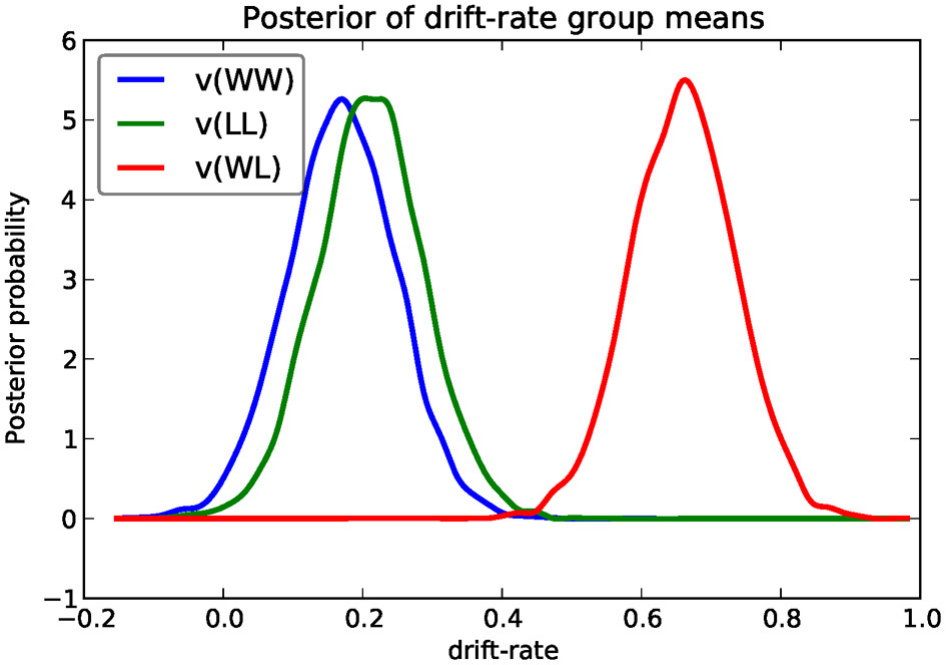
**Posterior density plot of the group means of the 3 different drift-rates *v* as produced by the hddm.analyze.plot_posterior_nodes() function**. Regions of high probability are more credible than those of low probability.

We next turn to comparing the posterior for the different drift-rate conditions. To plot the different traces we need to access the underlying node object. These are stored inside the nodes_db attribute which is a table (specifically, a DataFrame object as provided by the Pandas Python module) containing a row for each model parameter [e.g., v(WW)] and multiple columns containing various information about that parameter (e.g., the mean, or the node object). The node column used here represents the PyMC node object. Multiple assignment is then used to assign the 3 drift-rate nodes to separate variables. The hddm.analyze.plot_posterior_nodes() function takes a list of PyMC nodes and plots the density by interpolating the posterior histogram (see Figure 4).


___________________________________________

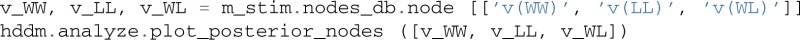





___________________________________________


Based on Figure 4 we might reason that the WL condition drift-rate is substantially greater than that for the other two conditions, which are fairly similar to each other.

One benefit of estimating the model in a Bayesian framework is that we can do significance testing directly on the posterior rather than relying on frequentist statistics (Lindley, 1965) (see also Kruschke (2010) for many examples of the advantages of this approach). For example, we might be interested in whether the drift-rate for WW is larger than that for LL, or whether drift-rate for LL is larger than WL. The below code computes the proportion of the posteriors in which the drift rate for one condition is greater than the other. It can be seen that the posteriors for LL do not overlap at all for WL, and thus the probability that LL is greater than WL should be near zero.

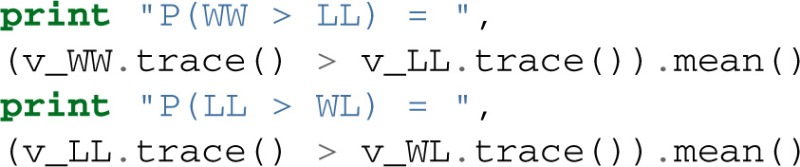



Which produces the following output.

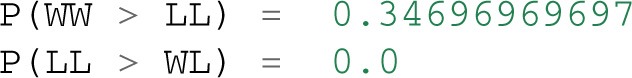



In addition to computing the overlap of the posterior distributions we can compare whether the added complexity of models with additional degrees of freedom is justified to account for the data using model selection. The deviance information criterion (Spiegelhalter et al., 2002) (DIC; lower is better) is a common method for assessing model fit in hierarchical models. The DIC is known to be somewhat biased in selecting the model with greater complexity, although alternative forms exist which improve this issue (see Plummer, 2008). Nevertheless, DIC can be a useful metric with this caveat in mind. One suggested approach is to generate simulated data from alternative models and use DIC to determine whether it properly selects the correct model given the same task contingencies. This exercise can help determine whether to rely on DIC, and also to provide an expected quantitative difference in DIC scores between models if one of them was correct, as a benchmark to compare DIC differences for fits to real data. We recommend interpreting significant differences in parameter estimates only within the models that fit the data the best penalized for complexity. By accessing the dic attribute of the model objects we can print the model comparison measure: 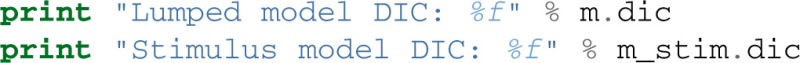


Which produces the following output:


Lumped model DIC: 10960.570932
Stimulus model DIC: 10775.615192


Based on the lower DIC score for the model allowing drift-rate to vary by stimulus condition we might conclude that it provides better fit than the model which forces the drift-rates to be equal, despite the increased complexity.

Note that Bayesian hypothesis testing and model comparison are areas of active research. One alternative to analyzing the posterior directly and the DIC score is the Bayes Factor (e.g., Wagenmakers et al., 2010).

### Fitting regression models

As mentioned above, cognitive neuroscience has embraced the DDM as it enables to link psychological processes to cognitive brain measures. The Cavanagh et al. (2011) study provides a useful illustration of the functionality. EEG recordings provided a trial-ty-trial measure of brain activity (frontal theta), and it was found that this activity correlated with increases in decision threshold in high conflict HF trials. Note that the data set and results exhibit more features than we consider here for the time being (specifically the manipulation of deep brain stimulation), but for illustrative purposes, we show only the code here to reproduce the main theta-threshold relationship in a model restricted to participants without brain stimulation. For more information, see Cavanagh et al. (2011).

The HDDMRegressor class allows individual parameters to be described by a linear model specification. In addition to the data argument, HDDMRegressor expects a linear model descriptor string to be provided. This descriptor contains the outcome variable that should be replaced with the output of the linear model—in this case a. The expression theta:C(stim) specifies an interaction between theta power and stimulus. The C() specifies that the stim column contains categorical data and will result in WL, LL, and WW being dummy coded. The Treatment argument encodes which condition should be used as the intercept. The two other conditions—LL and WW—will then be expressed *relative* to WL. For more information about the linear model specification syntax we refer to the Patsy documentation (patsy.readthedocs.org). In summary, by selecting data from the dbs off condition and specifying a linear model that uses categorical dummy-coding we can estimate a within-subject effect of theta power on threshold in different conditions.


___________________________________________

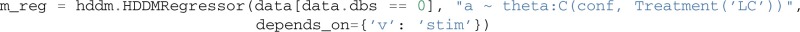




Which produces the following output:


Adding these covariates:
[’a_Intercept’, “a_theta:C(conf, Treatment('LC'))[HC]”,
                                “a_theta:C(conf, Treatment('LC'))[LC]”]
___________________________________________


Instead of estimating one static threshold per subject across trials, this model assumes the threshold to vary on each trial according to the linear model specified above (as a function of their measured theta activity). Cavanagh et al. (2011) illustrates that this brain/behavior relationship differs as a function of whether patients are on or off STN deep brain stimulation, as hypothesized by the model that STN is responsible for increasing the decision threshold when cortical theta rises).

**Figure 5:**
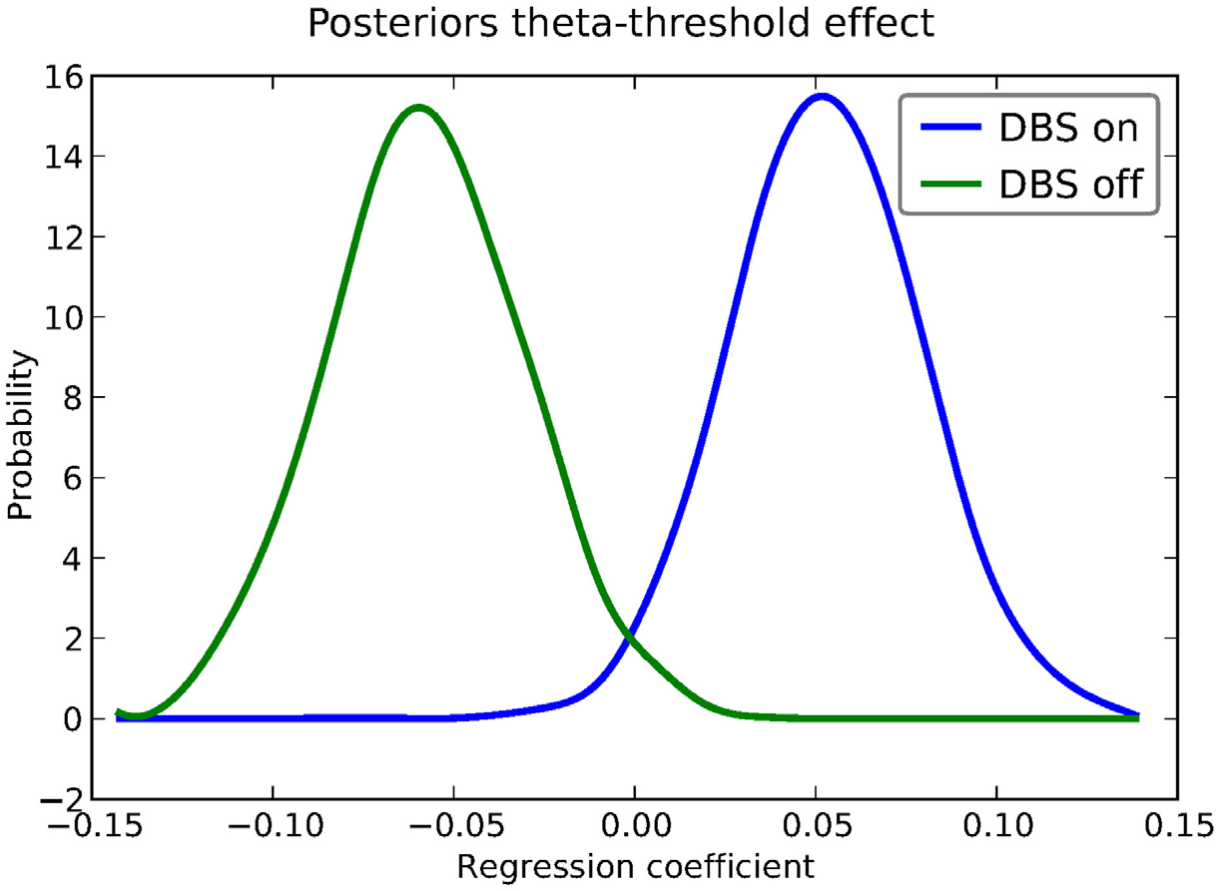
**Posterior density of the group theta regression coefficients on threshold *a* when DBS is turned on (blue) and off (green)**.

As noted above, this experiment also tested patients on deep brain stimulation (DBS). Figure 5 shows the regression coefficient of theta on threshold when the above model is estimated in the DBS off condition (in blue) and the DBS on condition (in green; code to estimate not shown). As can be seen, the influence of theta on threshold reverses. This exercise thus shows that HDDM can be used both to assess the influence of trial-by-trial brain measures on DDM parameters, but also how parameters vary when brain state is manipulated.

Finally, HDDM also supports modeling of within-subject effects as well as robustness to outliers. Descriptions and usage instructions of which can be found in the supplement.

## Simulations

To quantify the quality of the fit of our hierarchical Bayesian method we ran three simulation experiments. All code to replicate the simulation experiments can be found online at https://github.com/hddm-devs/HDDM-paper.

### Experiment 1 and 2 setup

For the first and second experiments, we simulated an experiment with two drift-rates (*v*_1_ and *v*_2_), and asked what the likelihood of detecting a drift rate difference is using each method. For the first experiment, we fixed the number of subjects at 12 (arbitrarily chosen), while manipulating the number of trials (20, 30, 40, 50, 75, 100, 150). For the second experiment, we fixed the number of trials at 75 (arbitrary chosen), while manipulating the number of subjects (8, 12, 16, 20, 24, 28).

For each experiment and each manipulated factor (subjects, trials), we generated 30 multi-subject data-sets by randomly sampling group parameters. For the first and second experiment, the group parameters were sampled from a uniform distribution 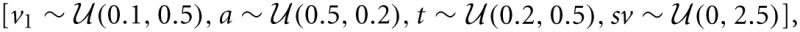 *sz* and *st* were set to zero, and *v*_2_ was set to 2*v_1_. To generate individual subject parameters, zero centered normally distributed noise was added to *v*_1_, *a*, *t*, and *sv*, with standard deviation of 0.2, 0.2, 0.1, and 0.1 respectively. The noise of *v*_2_ was identical to that of *v*_1_.

We compared four methods: (i) the hierarchical Bayesian model presented above with a within subject effect (HB); (ii) a non-hierarchical Bayesian model, which estimates each subject individually (nHB); (iii) the χ^2^-Quantile method on individual subjects (Ratcliff and Tuerlinckx, 2002); and (iv) maximum likelihood (ML) estimation using the Navarro and Fuss (2009) likelihood on individual subjects.

To investigate the difference in parameter recovery between the methods, we computed the mean absolute error of the recovery for each parameter and method in the trials experiment (we also computed this for the subjects experiment but results are qualitatively similar and omitted for brevity). We excluded the largest errors (5%) from our calculation for each method to avoid cases where unrealistic parameters were recovered (this happened only for ML and the quantiles method).

For each dataset and estimation method in the subject experiment we computed whether the drift-rate difference was detected (we also computed this for the trials experiment but results are qualitatively similar and omitted for brevity). For the non-hierarchical methods (ML, quantiles, nHB), a difference is detected if a paired *t*-test found a significant difference between the two drift-rates of the individuals (*p* < 0.05). For HB, we used Bayesian parameter estimation (Lindley, 1965; Kruschke, 2010). Specifically, we computed the 2.5 and 97.5 quantiles of the posterior of the group variable that models the difference between the two drift rates. An effect is detected if zero fell outside the quantiles. The detection likelihood for a given factor manipulation and estimation method was defined as the number of times an effect was detected divided by the total number of experiments.

### Experiment 3 setup

In the third experiment, we investigated the detection likelihood of trial-by-trial effects of a given covariate (e.g., a brain measure) on the drift-rate. We fixed the number of subjects at 12, and manipulated both the covariate effect-size (0.1, 0.3, 0.5) and the number of trials (20, 30, 40, 50, 75, 100, 150). To generate data, we first sample an auxiliary variable, α_*i*_ from 
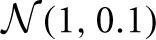
 for each subject *i*. We then sampled a drift-rate for each subject and each trial from 
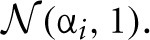
. The drift rate of each subject was set to be correlated to a standard normally distributed covariate (i.e., we generated correlated covariate data) according to the tested effect size. The rest of the variables were sampled as in the first experiments.

We compared all previous methods except the quantiles method, which cannot be used to estimate trial-by-trial effects. For the non-hierarchical methods (ML, quantiles, nHB), an effect is detected if a one sample *t*-test finds the covariate to be significantly different than zero (*p* < 0.05). For the HB estimation, we computed the 2.5 and 97.5 quantiles of the posterior of the group covariate variable. If zero fell outside the quantiles, then an effect was detected.

### Results

**Figure 6:**
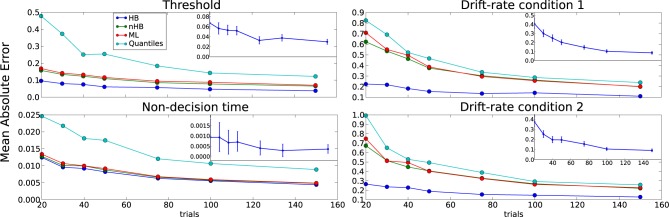
**Trials experiment**. Trimmed mean absolute error (MAE, after removing the 2.5 and 9.75 percentiles) as a function of trial number for each DDM parameter. Colors code for the different estimation methods (HB, Hierarchical Bayes; nHB, non-hierarchical Bayes; ML, maximum likelihood; and Quantiles, χ^2^-Quantile method). The inlay in the upper right corner of each subplot plots the difference of the MAEs between HB and ML, and the error-bars represent 95% confidence interval. HB provides a statistically significantly better parameter recovery than ML when the lower end of the error bar is above zero (as it is in each case, with largest effects on drift rate with few trials).

**Figure 7:**
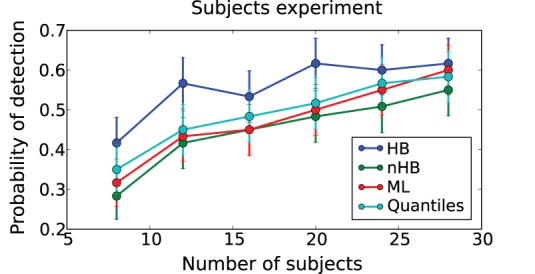
**Subjects experiment**. Probability of detecting a drift-rate difference (y-axis) for different numbers of subjects (x-axis) and different estimation methods (color coded; HB, Hierarchical Bayes; nHB, non-hierarchical Bayes; ML, maximum likelihood; and Quantiles, χ^2^-Quantile method). HB together with Bayesian hypothesis testing on the group posterior results in a consistently higher probability of detecting an effect.

**Figure 8:**
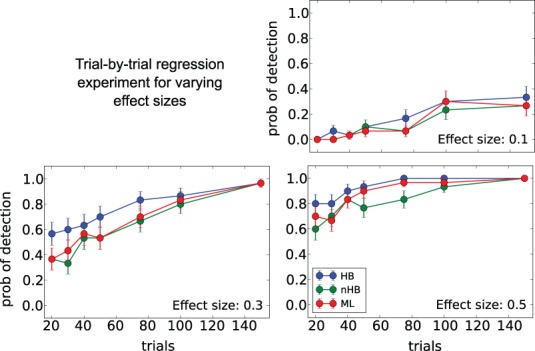
**Trial-by-trial covariate experiment**. Probability of detecting a trial-by-trial effect on drift-rate (y-axis) with effect-sizes 0.1 **(top left plot)**, 0.3 **(bottom left plot)** and 0.5 **(bottom right plot)** for different estimation methods (color coded; HB, Hierarchical Bayes; nHB, non-hierarchical Bayes; ML, maximum likelihood). While there is only a modest increase in detection rate with the smallest effect size, HB provides an increase in detection rate of up to 20% with larger effect sizes and fewer trials.

The detection likelihood results for the first experiment are very similar to the results of the second experiment, and were omitted for the sake of brevity. The HB method had the lowest recovery error and highest likelihood of detection in all experiments (Figures 6–8). The results clearly demonstrate the increased power the hierarchical model has over non-hierarchical ones. To validate that the increase in detection rate is not due to the different statistical test (Bayesian hypothesis testing compared to t-testing), but rather due to the hierarchical model itself, we also applied a *t*-test to the HB method. The likelihood of detection increased dramatically, which shows that the Bayesian hypothesis testing is not the source of the increase. However, the *t*-test results were omitted since the independence assumption of the test does not hold for parameters that are estimated using a hierarchical model.

The differences between the hierarchical and non-hierarchical methods in parameter recovery are mainly noticeable for the decision threshold and the two drift rates for every number of trials we tested, and it is most profound when the number of trials is very small (Figure 6). To verify that the HB method is significantly better than the other methods we chose to directly compare the recovery error achieved by the method in each single recovery to the recovery error achieved by the other methods for the same set dataset (inlay). For clarity purposes, we show only the comparison of HB with ML. The results clearly show that under all conditions HB outperforms the other methods.

## Discussion

Using data from our lab on a reward-based learning and decision-making task (Cavanagh et al., 2011) we demonstrate how HDDM can successfully be used to estimate differences in information processing based solely on RT and choice data. By using the HDDMRegression model we are able to not only quantify latent decision-making processes in individuals but also how these latent processes relate to brain measures (here theta power as measured by EEG had a positive effect on threshold) on a trial-by-trial basis. Critically, changing brain state via DBS revealed that the effect of theta power on threshold was reversed. As these trial-by-trial effects are often quite noisy, our hierarchical Bayesian approach facilitated the detection of this effect as demonstrated by our simulation studies (Figure 8), due to shared statistical structure among subjects in determining model parameters. This analysis is more informative than a straight behavioral relationship between brain activity and RT or accuracy alone. While we used EEG to measure brain activity this method should be easily extendable towards other techniques like fMRI (e.g., van Maanen et al., 2011). While trial-by-trial BOLD responses from an event-related study design are often very noisy, initial results in our lab were promising with this approach.

In a set of simulation studies we demonstrate that the hierarchical model estimation used in HDDM can recover parameters better than the commonly used alternatives (i.e. maximum likelihood and χ^2^-Quantile estimation). This benefit is largest with few number of trials (Figure 6) where the hierarchical model structure provides most constraint on individual subject parameter estimation. To provide a more applicable measure we also compared the probability of detecting a drift-rate and trial-by-trial effect and show favorable detection probability.

In conclusion, HDDM is a novel tool that allows researchers to study the neurocognitive implementations of psychological decision making processes. The hierarchical modeling provides power to detect even small correlations between brain activity and decision-making processes. Bayesian estimation supports the recent paradigm shift away from frequentist statistics for hypothesis testing (Lindley, 1965; Kruschke, 2010; Lee and Wagenmakers, 2013).

### Conflict of interest statement

The authors declare that the research was conducted in the absence of any commercial or financial relationships that could be construed as a potential conflict of interest.
