# Supplementary material for: HDDM: Hierarchical Bayesian estimation of the Drift-Diffusion Model in Python
Source: Front Neuroinform. 2013 Aug 2;7:14. doi: 10.3389/fninf.2013.00014 (PMC3731670; doi:10.3389/fninf.2013.00014)
Supplement: Supplementary file 1 [file Table1.PDF]

HDDM: Hierarchical Bayesian estimation of the Drift-Diffusion Model in Python – Supplemental Material

Thomas V. Wiecki*, Imri Sofer*, Michael J. Frank

May 6, 2013

The code to replicate the analyses performed in this paper can be downloaded as an IPython notebook from this address:

http://nbviewer.ipython.org/urls/raw.github.com/hddm-devs/hddm/develop/hddm/examples/hddm_demo.ipynb.

Hierarchical Bayesian Estimation

Bayesian methods require specification of a generative process in form of a likelihood function that produced the observed data x given some parameters θ. By specifying our prior beliefs (which can be informed or non-informed) we can use Bayes formula to invert the generative model and make inference on the probability of parameters θ:

$$P(\theta|x) = \frac{P(x|\theta) \times P(\theta)}{P(x)},$$

where $P(x|\theta)$ is the likelihood of observing the data (in this case choices and RTs) given each parameter value and $P(\theta)$ is the prior probability of the parameters. In most cases the computation of the denominator is quite complicated and requires to compute an analytically intractable integral. Sampling methods like Markov-Chain Monte Carlo (MCMC) (Gamerman and Lopes, 2006) circumvent this problem by providing a way to produce samples from the posterior distribution. These methods have been used with great success in many different scenarios (Gelman et al., 2003) and will be discussed in more detail below.

As noted above, the Bayesian method lends itself naturally to a hierarchical design. In such a design, parameters of a distribution can themselves be expressed as random variables with their own priors. This hierarchical property has a particular benefit to cognitive modeling where data is often scarce: We can construct a hierarchical model that explicitly captures the similarity between individual's parameter values. As above, observed data points of each subject $x_{i,j}$ (where $i = 1, \ldots, S_j$ data points per subject and $j = 1, \ldots, N$ for N subjects) are distributed according to some likelihood function $f(\theta)$. We now assume that individual subject parameters θ_j are normally distributed around a group mean μ with a specific group standard deviation σ, resulting in the following generative description:

$$\theta_j \sim \mathcal{N}(\mu, \sigma^2)$$
$$x_{i,j} \sim f(\theta_j)$$

Priors

The informative group mean priors are created to roughly match parameter values reported in the literature and collected by (Matzke and Wagenmakers, 2009). Figure 1 shows the the prior probability densities used for each group mean parameter on top of the empirical values reported in the literature.

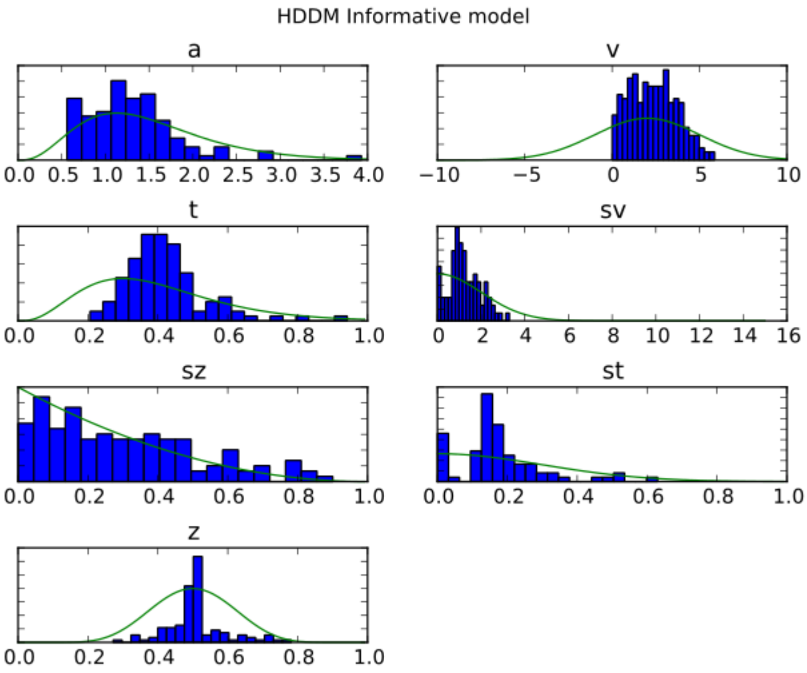

Figure 1: Green: Probability densities of prior distributions for group means of each DDM parameter of the informative HDDM model. Blue: Histogram over DDM parameters reported in the literature (Matzke and Wagenmakers, 2009).

Reaction Time distributions

To provide the reader with a sense of how RT data is distributed we plot histograms of each individual's RTs in figure 2. Because there are two possible responses (here we are using accuracy coding where 1 means the more rewarding symbol was chosen, and 0 the less rewarding) we flip error RTs to be negative. The following code was used to generate the RT histogram plot and uses `Pandas`' `groupby` functionality which splits the data set into parts containing individual subject data.

```
data = hddm.utils.flip_errors(data)

fig = plt.figure()
ax = fig.add_subplot(111, xlabel='RT', ylabel='count', title='RT distributions')
for i, subj_data in data.groupby('subj_idx'):
    ax.hist(subj_data.rt, bins=20, histtype='step')
```

Within-subject effects

In the main manuscript we estimated a between-subject difference in drift-rate for the three different conditions – `LL`, `WW` and `WL`. Note that this model implicitly assumes that the different conditions are completely independent: each drift rate was sampled from a separate group prior. However, there may be individual differences in overall performance, and if so it is reasonable to assume that someone who would be better at `WL` would also be better at `LL`. To model this intuition we can use a within-subject model where an intercept is used to capture overall performance in the `WL` condition as a baseline, and then the other `LL` and `WW` conditions are expressed relative to `WL`. (Perhaps every subject has a higher drift in `WL` than `LL` but there is huge variance in their overall drift rates. In this scenario, the earlier model would not have the power to detect the effect of condition on this within subject effect, because there would be large posterior variance in all of the drift rates, which would then overlap with each other. In contrast, the within-subject

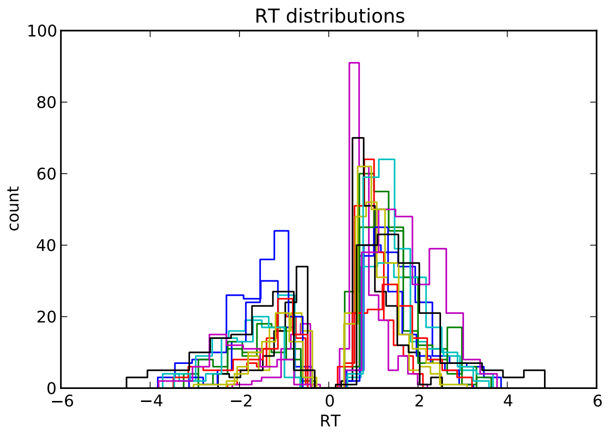

Figure 2: Reaction time histograms of individual subjects. Reaction time is distributed along the x-axis. Error responses are mirrored on the y-axis and thus appear negative. Bin-count is distributed along the y-axis.

model would estimate large variance in the intercept but still allow the model to infer a non-zero effect of condition with high precision).

To estimate a within-subject effect we can use the `HDDMRegressor` class we already used to estimate the trial-by-trial correlations. Internally, `HDDMRegressor` uses the `Patsy` module to construct a design matrix from the linear model descriptor, see https://patsy.readthedocs.org/en/latest/ for more details.

```
m_within_subj = hddm.HDDMRegressor(data, "v ~ C(stim, Treatment('WL'))")
```

Which outputs the newly created covariates which will be used in the regression:

```
Adding these covariates:
['v_Intercept', "v_C(stim, Treatment('WL'))[T.LL]", "v_C(stim, Treatment('WL'))[T.WW]"]
```

As alluded to above, `WL` will be used as the intercept. `v_C(stim, Treatment('WL'))[T.LL]` and `v_C(stim, Treatment('WL'))[T.WW]` are new parameters that will be estimated relative to the intercept.

To estimate the model we again draw samples from the posterior. Note that regression models are often slower to converge so we use more burn-in and draw more samples to accommodate this fact.

```
m_within_subj.sample(10000, burn=1000)
```

To examine the marginal posteriors of our individual regressors we can again plot them using the following code (see figure 3 for the output):

```
v_WL, v_LL, v_WW = m_within_subj.nodes_db.node[["v_Intercept",
                                                "v_C(stim, Treatment('WL'))[T.LL]",
                                                "v_C(stim, Treatment('WL'))[T.WW]"]]
hddm.analyze.plot_posterior_nodes([v_WL, v_LL, v_WW])
```

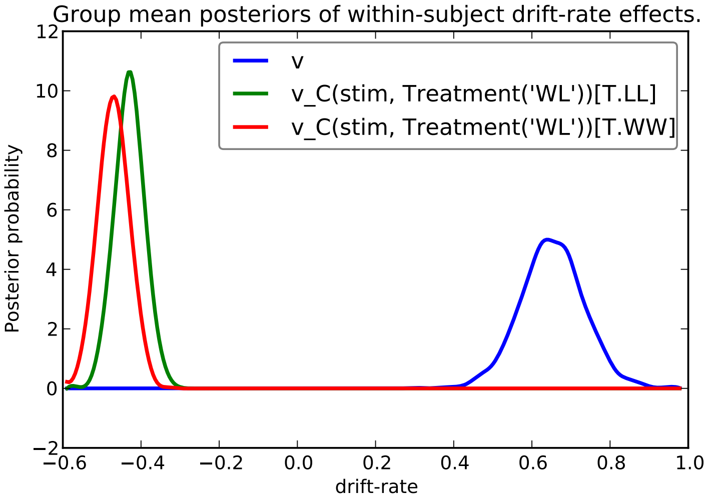

Figure 3: Posterior density plot of the group means of the 3 different within-subject drift-rates v as produced by the `hddm.analyze.plot_posterior_nodes()` function. Note that while v (blue line) represents the drift-rate of the `WL` condition, the other two posteriors (green and red lines) are expressed relative to this condition.

Note that in the above plot `LL` and `WW` are expressed relative to the `WL` condition (i.e. `v_Intercept`). Thus, while the overall drift rate intercept, here applying to the `WL` condition, is positive (mode value roughly 0.7), the relative within subject effects of condition (`WW` and `LL`) are negative and do not overlap with zero – suggesting a significant effect of this condition on drift-rate.

Dealing with outliers

It is common to have outliers in any data set and RT data are no exception. Outliers present a serious challenge to likelihood-based approaches, as used in HDDM. Consider the possibility that 5% of trials are not generated by the DDM process, but by some other process (e.g. due to an attentional lapse). The observed data in those trials may be very unlikely given the best DDM parameters that fit 95% of the data. In the extreme case, the likelihood of a single trial may be zero (e.g. if subjects respond very quickly, faster than the non-decision time `t` parameter that would fit the rest of the data). Thus this single outlier would force the DDM parameters to adjust substantially. To illustrate the effect of this we will generate data with outliers, but fit a standard DDM model without taking outliers into account.

```
outlier_data, params = hddm.generate.gen_rand_data(params={'a': 2, 't': .4, 'v': .5},
                                                   size=200, n_fast_outliers=10)

m_no_outlier = hddm.HDDM(outlier_data)
m_no_outlier.sample(2000, burn=50)

m_no_outlier.plot_posterior_predictive()
```

As the left plot of figure 4 shows, the predictive likelihood does not fit the RT data very well. The model predicts far more fast RTs than are actually observed. This is because non-decision time `t` is forced to be estimated small enough to account for a few fast RTs.

To solve this issue, HDDM includes a mixture model which assumes that outliers come from a uniform distribution. Here, we specify that we expect roughly 5% outliers in our data.

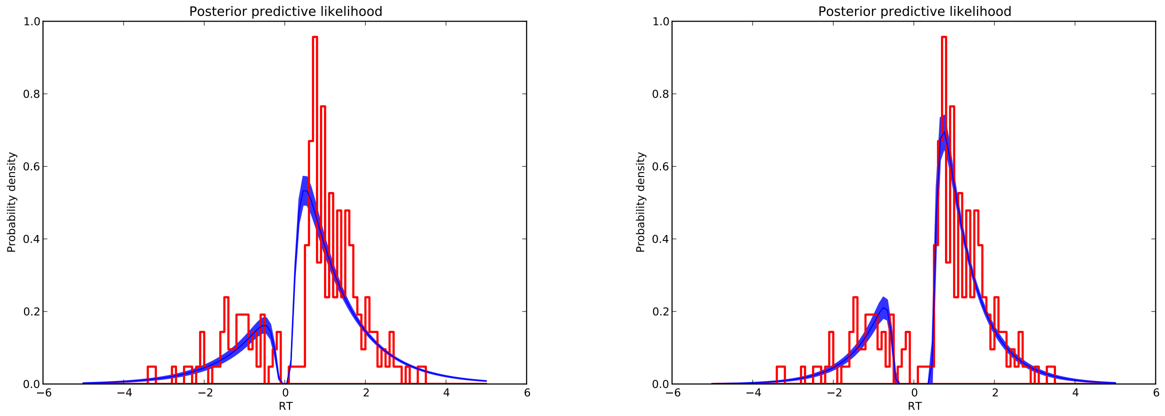

Figure 4: Posterior predictive likelihood of the model which does not allow for outliers (left) and the model which does allow for outliers (right). Error responses are mirrored along the y-axis. Histogram of reaction times in red and posterior predictive likelihood in blue. Time increases along the x-axis while probability density is plotted against the y-axis.

```
m_outlier = hddm.HDDM(outlier_data, p_outlier=.05)
m_outlier.sample(2000, burn=20)

m_outlier.plot_posterior_predictive()
```

The mixture model provides a much better fit (see the right plot in figure 4) because outlier RTs are having less of an effect because they get assigned to the uniform outlier distribution. Note that it is also possible to estimate the probability of obtaining outliers from the data by setting `include=['p_outlier']` instead of `p_outlier=x` when `HDDM` in instantiated.

References

Gamerman, D. and Lopes, H. (2006). *Markov chain Monte Carlo: stochastic simulation for Bayesian inference*. Chapman \& Hall/CRC.

Gelman, A., Carlin, J., Stern, H., and Rubin, D. (2003). *Bayesian data analysis*. Chapman \& Hall/CRC.

Matzke, D. and Wagenmakers, E. (2009). Psychological interpretation of the ex-Gaussian and shifted Wald parameters: A diffusion model analysis. *Psychonomic Bulletin & Review*.
